# Letrozole or clomiphene citrate as first line for anovulatory infertility: a debate

**DOI:** 10.1186/1477-7827-9-86

**Published:** 2011-06-21

**Authors:** Mohan S Kamath, Korula George

**Affiliations:** 1 Reproductive Medicine Unit, Christian Medical College Hospital, Vellore 632004, Tamil Nadu, India

## Abstract

Clomiphene citrate has been traditionally used as the drug of choice in treating women with anovulatory infertility. In the last decade letrozole, an aromatase inhibitor has emerged as alternative ovulation induction agent. Literature confirms that letrozole has a definitive role in anovulatory women who have not responded to the clomiphene therapy. However its role as an alternative to clomiphene as first line therapy continues to be debated. Although it is probable that the overall benefits of letrozole surpass clomiphene citrate, currently available data does not confirm this view. There is need for large well-designed trials.

## Background

Anovulatory dysfunction is a common problem and is responsible for about 40% of female infertility. Polycystic ovarian syndrome (PCOS) remains one of its leading causes [1]. Using the Rotterdam criteria a clinical diagnosis of PCOS is easily reached and most often treatment can be initiated following a few basic investigations and exclusion of a male factor problem.

Clomiphene citrate is considered as the drug of choice for first line treatment of anovulatory dysfunction for a variety of reasons. It is orally administered, has few side effects, is easily available and is inexpensive. Although ovulation rates are in the range of 70-80% the actual pregnancy rates are significantly lower at around 30-40% [1,2]. Clomiphene resistance together with side effects like multi-follicular development and cyst formation are areas of concern. The desire for an effective alternative persists.

Letrozole, an aromatase inhibitor, was introduced into infertility practice in the year 2000 and is regarded as a second line treatment option, particularly in women with clomiphene resistance [3,4]. Letrozole has found acceptance in various clinical situations and the indications for use have expanded [5,6]. In contrast to clomiphene, letrozole at the customary dose of 2.5 mg elicits a monofollicular response and does not adversely affect either the endometrium or the cervical mucus, due to an absence of a peripheral estrogen receptor blockage. The oft asked question of whether it is better than clomiphene as a first line treatment option remains unanswered and a clear answer would have important clinical implications for infertility specialists.

## Clomiphene vs. letrozole: mechanism of action

Clomiphene, a non steroidal compound, structurally similar to estrogen, blocks estrogenic hypothalamic receptors, resulting in blinding of the hypothalamus-pituitary axis to endogenous circulating estrogen. This in turn triggers release of FSH from the anterior pituitary following alterations in GnRH pulsatility. Clomiphene also has peripheral anti estrogenic action at the level of the endometrium and cervical mucus, partly explaining the discrepancy in ovulation rates and pregnancy rates [1].

Letrozole, a selective aromatase inhibitor, prevents the conversion of androgens to estrogen, thus releasing the hypothalamo-pituitary axis from the negative feed back of estrogen, resulting in an increase of FSH secretion from the anterior pituitary. The accumulated androgens in the ovary further increase follicular sensitivity to FSH [7].

Importantly, unlike clomiphen citrate, letrozole is devoid of any anti estrogenic peripheral action. Letrozole is also cleared from the circulation more rapidly due to a shorter half life (48 hours) as compared to clomiphene citrate which may take up to 2 months due to its prolonged half life (2 weeks) [8].

## First line therapy for anovulation - clomiphene citrate vs letrozole

Several studies have looked at letrozole versus clomiphene as first line therapy in anovulatory infertility.

Atay V et al randomized 106 women with PCOS (55/51) to receive either letrozole (2.5 mgs) or clomiphene citrate (100 mgs/day) [9]. The ovulation rate (82.4% Vs 63.6%, *P *= 0.01) and the clinical pregnancy rate (21.6% vs. 9.1%, *P *= 0.03) were significantly higher in the letrozole group as compared to the clomiphene group with the authors recommending letrozole as a better first line approach. Although a randomized trial, details regarding method of randomization, allocation concealment and sample size calculation are not available, thus raising concerns regarding reliability.

In another randomized controlled trial (RCT) Bayar U et al compared letrozole versus clomiphene citrate as a first line ovulation inducing agents [10]. There was no significant difference in either the ovulation rate or the clinical pregnancy rate between the two groups [(65.7% vs 74.7%) and (9.1% vs 7.4%)]. While well designed, the sample size was calculated using the projected difference in follicular numbers and estrogen concentrations on the day of hCG administration. The authors favor use of letrozole as first line therapy due to comparable clinical pregnancy rates and the advantage of development of fewer follicles, thus reducing the risk of multiple pregnancies.

In the largest RCT trial involving 438 women with PCOS, Badawy et al compared letrozole versus clomiphene [11]. Details regarding allocation concealment or sample size calculation are not mentioned and the designated primary outcome was the number of developed follicles, hormone levels and endometrial thickness on the day of hCG administration. With the ovulation and pregnancy rates being comparable between the two groups, the authors concluded that no benefit was observed with the use of letrozole as first line therapy, especially since the cost of the drug is comparatively high. Interestingly the endometrial thickness in the women who received clomiphene was significantly higher than in the Letrozole group, (9.2 +- 0.7 Vs 8.1 +-0.2 mm, P = 0.02), which is contrary to the traditionally accepted view (Table 1).

**Table 1:** RCTs comparing letrozole versus clomiphene as first line for anovulatory women

Sl no	Authors	Study design	Treatment arms	Numbers cycles	Endometrial thickness (mm)	Ovulation rates (%)	Pregnancy rates (%)
1	Atay V et al. [9] Turkey, 2006	RCT	Letrozole 2.5 mg vs. Clomiphene 100 mg	51 vs. 55	8.4 +/- 1.8 vs. 5.2 +/- 1.2	82.4 vs. 63.6	21.6 vs. 9.1
2	Bayar U et al. [10] Turkey, 2006	RCT	Letrozole 2.5 mg vs. Clomiphene 100 mg	99 vs. 95	8# vs. 8#	65.7 vs. 74.7	9.1 vs. 7.4
3	Badawy A et al. [11] Egypt, 2009	RCT	Letrozole 5 mg vs. Clomiphene 100 mg	540 vs. 523	8.1+/-0.2 vs. 9.2+/-0.7	67.5 vs. 70.9	15.1 vs. 17.9

# Values represent median

Begum et al studied a different category of women, recruiting for their RCT women who did not respond to 100 mg of clomiphene [12]. The study group received 7.5 mgs of letrozole while the control group was given clomiphene citrate at a dose of 150 mg. Not unexpectedly, the ovulation rates in the letrozole arm were significantly higher as compared to the clomiphene arm (62.5% vs 37.5%). The results of this trial do not help in answering the specific issue in question, as the cohort of women studied possibly represent a clomiphene resistant group.

In their meta-analysis, Polyzos et al reviewed the role of aromatase inhibitors in female infertility, summing up the presently available literature [13,14]. The pooled data from 4 randomized trials (Atay et al 2006; Bayar et al 2006; Sorabvand et al 2006; Sipe et al 2006) involving 265 women with PCOS revealed a significantly higher live birth rate per patient with aromatase inhibitors as compared to clomiphene citrate (OR 2.4, 95% CI 1.2-4.6, *P *= .011.) [9,10,13,15,16].Only two of the included studies specifically compared letrozole and clomiphene as a single agent therapy. Although homogenous, the included studies were small in numbers. The results of the largest trial by Badawy et al, which was not included in the meta-analysis, do not match the conclusion of the meta-analysis [11,13].

Requena et al in their literature review looked at randomized trials comparing letrozole versus clomiphene as first line therapy and included four studies (Atay et al 2006; Bayar et al 2006; Sorabvand et al 2006; Badawy et al 2007) [9-11,15,17]. The ovulation rate for letrozole in comparison with clomiphene did not differ significantly (OR 1.7; 95% CI 0.66 - 2.09) nor did the pregnancy rate per patient (OR 1.37; 95% CI 0.70 - 2.71). However results need to be interpreted cautiously since the studies included were not statistically homogenous (*I *^*2 *^>50%).

A review of the clinical trial registry indicates that several trials reviewing the role of letrozole as an ovulatory inducing agent are underway and hopefully these will help in arriving at firmer conclusions [18].

## Clomiphene resistance

The options for women not responding to increasing doses of clomiphene include insulin sensitizers, gonadotrophins, or laparoscopic ovarian drilling [19,20]. Mitwally and Casper in a non randomized study evaluated letrozole as an alternative and obtained an ovulation rate of 75% [3].

In an RCT comparing letrozole versus anastrazole in clomiphene resistant women Al Omari et al achieved an ovulation rate of 84% while Elnashar et al in a comparative study obtained an ovulation rate of 54% using letrozole [4,21]. We conducted a randomized double blind placebo controlled trial using letrozole 2.5 mgs in clomiphene resistant women and obtained an ovulation rate of 33.3% [22]. The discrepancy in the ovulation rates could be attributed to either the dose of the drug chosen or to the small numbers studied. An expected response would be in the range of 30-50% in this group of women. Letrozole was used as an adjunct for breast cancer treatment at a dose 2.5 mgs. A similar dose was followed for ovulation induction.

Al Fahidi et al, looking at different dosage schedules (5 mgs vs. 2.5 mgs), found a significant increase in the number of mature follicles and the pregnancy rate/cycle with a dose of 5 mg. No multiple pregnancies were recorded in either group [23].

Summing up, Letrozole appears to an effective ovulation inducing agent in women with clomiphene resistance, though dosing issues still need to be resolved.

## Clomiphene failure

Quentero et al looked at the options available for women who failed to conceive despite ovulation following clomiphene therapy (clomiphene failure) [24]. In a small group of such women they compared letrozole versus gonadotrophins and obtained significantly higher pregnancy rates with gonadotrophins (28% vs. 9%). There were 2 multiple pregnancies in the gonadotrophin group as compared to none in the letrozole group. In comparison to gonadotrophins, letrozole is less expensive and requires less intense cycle monitoring, thus making it an option for women with clomiphene failure.

Looking at the data from a different angle, additional pregnancies achieved in this group (CC failure) and in clomiphene resistant women, could be a possible explanation for the higher pregnancy rates obtained using letrozole as a first line in one of the earlier study [9]. One can always argue that the reverse could also be true: a group of anovulatory women with letrozole failure/resistance who may respond to clomiphene. Currently we do not have data to substantiate this argument. However first line use of letrozole will eventually generate information to answer such questions.

## Time to pregnancy

An issue of great importance to couples seeking infertility treatment is the time to pregnancy. Clinicians usually follow a step ladder approach in advising treatment: starting with the simplest and least expensive mode of therapy and then moving higher as required. However in certain clinical situations like advanced female age or severe endometriosis starting at a higher level would be more appropriate.

When clomiphene citrate is used as first line therapy in anovulatory women one can expect a 25% incidence of clomiphene resistance. It has been established that although ovulation rates are in the range of 75% only 30-40% will actually conceive [1]. Hence about 60-65% of anovulatory women being treated with clomiphene will fall into either the resistant or failure group. However, conventionally one starts with a lower dose of clomiphene citrate, gradually building up the dose. Hence a diagnosis of either of these conditions is arrived at only after several months of therapy. Letrozole has been shown to be effective in women with either clomiphene resistance or failure [4,24]. As pointed out by R. Casper using letrozole as first line therapy would logically help women bypass these problems thus avoiding superfluous treatment cycles, which would be especially useful in older women [25]. Intangible benefits are difficult to quantify or document but nevertheless are of importance to couples.

Letrozole has now been in use as an ovulation induction agent for more than a decade. Even though emerging evidence suggests that it is an effective ovulation induction agent, comparable if not better than clomiphene, it has still not gained universal acceptance for a variety of reasons. An abstract presentation at ASRM 2005 suggested an increase in congenital malformations following letrozole treatment. The attention generated by this publication resulted in the manufacturers (Novartis) declaring that use of letrozole as an ovulation induction agent is contraindicated [26]. These observations were critically analyzed by Tulandi et al who presented additional data, assuaging fears regarding safety of letrozole [27].

However the worries and concerns continue and today letrozole has still not received approval for use in fertility treatment in the United States, Europe and many other parts of the world. Apart from denying anovulatory women access to a less expensive, simpler and effective treatment option, it has lead to a dearth of well designed trials assessing its role in infertility. Though some evidence is currently available larger well designed trials are required to pave the way forward for wider use.

## Conclusions

To conclude letrozole has a definitive role to play in women with either clomiphene failure or resistance. Currently available data comparing clomiphene and letrozole as a first line therapy for women with anovulation is conflicting in nature and inconclusive. It is possible that the overall benefits of letrozole may surpass clomiphene citrate as it could be beneficial in a subgroup of women who may not successfully respond to clomiphene either due to resistance or failure. However there is need for larger well designed randomized trials to generate robust data in order to establish the true potential of letrozole.

## Competing interests

The authors declare that they have no competing interests.

## Authors' contributions

Both the authors MSK and KG conceived the report, drafted the manuscript, designed the Table, and carried the literature search. Both authors read and approved the final manuscript.
